# Safety of Knee Arthroplasty Following Genicular Artery Embolization for Knee Osteoarthritis

**DOI:** 10.1007/s00270-025-04227-z

**Published:** 2025-10-15

**Authors:** Helen Albrecht, Lucas R. Cusumano, Mark Little, Erik N. Zeegen, Ahmad Al-rekabi, Nev Davies, Siddharth A. Padia

**Affiliations:** 1 University of California, Los Angeles, USA; 2 Section of Interventional Radiology, David Geffen School of Medicine, University of California Los Angeles, 757 Westwood Plaza, Suite 2125, Los Angeles, CA 90095 USA; 3 Department of Radiology, Royal Berkshire NHS Foundation Trust, Reading, UK; 4 Department of Orthopaedic Surgery, David Geffen School of Medicine, University of California Los Angeles, Los Angeles, CA USA; 5 Department of Orthopaedic Surgery, Royal Berkshire NHS Foundation Trust, Reading, UK

**Keywords:** GAE, Genicular artery embolization, KA, Knee arthroplasty, PKA, Partial knee arthroplasty, TKA, Total knee arthroplasty, Knee, Pain, Osteoarthritis

## Abstract

**Purpose:**

Concerns may exist as to potential healing complications after knee arthroplasty (KA) following genicular artery embolization (GAE) for knee osteoarthritis (OA). This study evaluates the adverse events related to KA following GAE.

**Materials and Methods:**

This IRB-approved multi-institution retrospective study (2019–2024) analyzed 47 patients who underwent 48 KA’s following 48 GAEs (out of a total of 300 GAEs).. Post KA records were reviewed for the presence of adverse events.

**Results:**

KA was performed by one of 16 orthopedic surgeons at 10 hospitals. Mean time between GAE and KA was 512 ± 330 days. There were 39/48 (81.2%) TKAs and 9/48 (18.8%) partial KAs. Follow-up time after KA was a mean of 369 (± 350) days. Four (4/48 = 8.3%) AEs were reported including two (2/48 = 4.2%) cases of wound healing issues, a stitch abscess and non-infected fluid discharge at the surgical incision. All AEs resolved. There were no significant differences between mean age (64.3 ± 4.2 vs. 68.7 ± 10.7 years, P = 0.239), BMI (25.4 ± 2.67 vs. 27.9 ± 5.2, P = 0.324), number of arteries treated (2.0 ± 0.8 vs. 1.8 ± 0.7, P = 0.693), and time between GAE and KA (491.8 ± 416.5 vs 513.4 ± 326.6 days, P = 0.787) between groups with and without AEs, respectively.

**Conclusion:**

Adverse events related to GAE for patients undergoing KA are low. GAE should not be considered a contraindication for future KA and may serve as a bridge in those trying to defer KA to a future date.

**Graphical Abstract:**

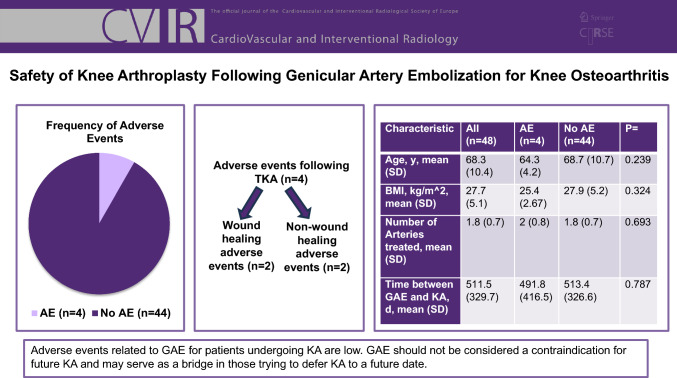

## Introduction

Knee arthroplasty (KA) is a well-established and effective treatment for advanced knee osteoarthritis (OA) [1]. However, it carries a measurable risk of adverse events (AEs)—typically 5–6% for major complications within 30 days, with minor complications also contributing to postoperative morbidity [2]. Common AEs include wound complications, prosthetic joint infection, thromboembolic events, periprosthetic fractures, stiffness, and aseptic loosening [3]. These complications may arise from patient-related risk factors (e.g., diabetes, obesity, peripheral vascular disease, impaired healing capacity, history of previous surgery) or surgical factors [2, 4, 5].

Genicular artery embolization (GAE) has arisen as a promising minimally invasive option for pain management associated with OA that alleviates symptoms in patients with OA via reducing synovial arterial hypervascularity [6, 7]. GAE offers a viable alternative for managing OA-related pain, particularly for patients who are not currently suitable candidates for KA. [8] While some may undergo GAE in lieu of KA, others may use GAE in order to delay KA until a more appropriate time. However, the impact of GAE on subsequent surgical outcomes remains poorly understood.

The rationale for concern is that GAE, by reducing blood supply to synovium and periarticular tissues, could theoretically impair wound healing, delay tissue repair, or increase the risk of wound dehiscence after KA. In addition, non-target embolization to skin or bone could predispose to ischemic changes or necrosis [7, 9, 10] raising the possibility of higher rates of infectious complications or poor osseointegration of implants. These potential mechanisms suggest that prior GAE may alter the risk profile of KA beyond standard healing concerns.

Given the growing adoption of GAE in the OA treatment pathway, it is critical to understand whether this procedure increases the likelihood of complications after KA. The objective of this study was to evaluate the safety of KA following GAE as a treatment for symptoms associated with OA.

## Materials and Methods

### Patients

This multi-institution retrospective study was approved by each center’s institutional review board, with waiver of informed consent. From January 2019 until July 2024, a total of 300 GAEs were performed for symptomatic knee OA. Clinical response after GAE was reported in 86 of the 300 patients in two separate prospective trials [7, 11]. GAE was performed in patients who met the following inclusion criteria: symptomatic knee OA, ineligibility or refusal of KA, and failure of conservative treatments (e.g. steroid injection, physical therapy, and nonsteroidal anti-inflammatory drugs). The most common reason to defer KA at the time of GAE was concerns regarding recovery time and commitment to rehabilitation.

### GAE technique

GAE was performed by one of six board certified interventional radiologists using previously described techniques.*

Briefly, in 39 out of the 48 patients radiopaque skin markers were placed at the site(s) of knee pain prior to the procedure. The remaining 9 patients had treatment based on patient symptoms and MRI findings. In all patients, ultrasound guided ipsilateral or contralateral common femoral artery access was performed. A digital subtraction angiogram (DSA) performed with the catheter in the ipsilateral superficial femoral artery was utilized to identify areas of hypervascularity. Cone-beam computed tomography (CBCT) was either performed at this location and/or following genicular artery catheterization with using a 1.7 Fr to 2.4 Fr microcatheter. Nitroglycerin was injected through the microcatheter to optimize antegrade flow to the target. Embolization was performed with 100 µm Embozene particles (Varian Medical Systems, Palo Alto, California) or 100–300 µm Embosphere particles (Merit Medical Systems, Inc, South Jordan, Utah). The embolization endpoint was pruning of abnormal hyperemia with preservation of the normal arterial flow. Following embolization of all target arteries, hemostasis was achieved with manual compression for antegrade access or arterial closure device for contralateral access. Patients were discharged four hours following the procedure.

Outpatient clinical assessments were performed periodically, starting at one month following GAE with continued clinic follow-up for at least one year.

### Knee Arthroplasty Procedures and Follow-Up

Records were reviewed for all patients who underwent GAE for incidence of KA. Operative reports were reviewed to distinguish between total and partial KA. Partial KA was further subdivided into knee compartment (i.e. medial or lateral) and if this compartment was the same compartment treated during GAE. Operative reports and post-procedure clinical records were analyzed for the presence of AEs. AEs were then characterized as either wound healing related (e.g. delayed wound healing, wound dehiscence, and presence of tissue necrosis) or non-wound healing related (e.g. infections, bleeding, venous thrombosis, and prosthetic failure).

### Statistical Analysis

Continuous data are expressed as mean ± standard deviation. Comparisons between groups were made using the Fisher exact or Chi-square test for categorical variables and analysis of variance (ANOVA) or the Mann–Whitney analysis for continuous variables. *P* values < 0.05 were considered significant. Statistical analyses were performed using a computer using GraphPad Prism, version 9.3 (GraphPad Software, La Jolla, California, USA).

## Results

### Patient Characteristics

The mean patient age was 68.3 ± 10.4 years old with a mean body mass index of 27.7 ± 5.1 kg/m^2^. The procedure was performed on 26 (54.1%) right knees and 22 (45.8%) left knees. The breakdown of the types of arteries embolized during GAE are as follows; descending genicular artery was embolized in 58.3% (28/48) of patients, superior medial artery was embolized in 29.2% (14/48) of patients, the inferior medial artery was embolized in 39.6% (19/48) of patients, the superior lateral artery was embolized in 16.7% (8/48) of patients and the inferior lateral artery was embolized in 29.2% (14/48) of patients. Knee OA severity as determined by Kellgren -Lawrence (KL) score ranged from 1 to 4 with a median KL score of 3. A mean of 1.8 ± 0.7 genicular arteries were embolized per procedure (Table 1). Volume of embolic agent was relatively consistent throughout the patient cohort (2–5% of a single vial per embolized artery).

**Table 1:** Patient demographics and procedural factors

Characteristic	All (n = 48)	AE (n = 4)	No AE (n = 44)	P =
Age, year, mean (SD)	68.3 (10.4)	64.3 (4.2)	68.7 (10.7)	0.239
BMI, kg/m^2^, mean (SD)	27.7 (5.1)	25.4 (2.67)	27.9 (5.2)	0.324
Number of arteries treated, mean (SD)	1.8 (0.7)	2 (0.8)	1.8 (0.7)	0.693
*Arteries embolized*				
Descending genicular	28/48 (58.3%)	2/4 (50.0%)	26/44 (59.1%)	0.182
Superior medial	14/48 (29.2%)	1/4 (25.0%)	13/44 (29.5%)	0.852
Inferior medial	19/48 (39.6%)	1/4 (25.0%)	18/44 (40.9%)	0.543
Superior lateral	8/48 (16.7%)	1/4 (25.0%)	7/44 (15.9%)	0.649
Inferior lateral	14/48 (29.2%)	2/4 (50.0%)	12/44 (27.3%)	0.349
Time between genicular artery embolization (GAE) and knee arthroplasty (KA), day, mean (SD)	511.5 (329.7)	491.8 (416.5)	513.4 (326.6)	0.787
Knee laterality				
Left	22/48 (45.8%)	3 (75%)	19 (43%)	
Right	26/48 (54.1%)	1 (25%)	25 (57%)	0.320
*Kellgren–Lawrence score*				
1	1/48 (2.1%)	0	1/44 (2.3%)	
2	6/48 (12.5%)	0	6/44 (13.6%)	
3	32/48 (66.7%)	3/4 (75%)	29/44 (65.9%)	
4	9/48 (20.5%)	1/4 (25%)	8/44 (18.2%)	0.856
*Embolic*				
Embosphere	9/48 (18.8%)	4/4 (100%)	35/44 (80%)	

Values are reported as mean (SD) or n (%)

### Knee Arthroplasty Surgeries

KA was performed in 48 knees following GAE by 16 orthopedic surgeons at 10 different hospitals. 45 (93.8%) total and three (6.3%) partial KAs were performed. All of the partial KAs were performed in the medial compartment, which was also the same compartment treated with GAE. Post surgical follow-up records were available after 40 (83.3%) KAs. Mean time between GAE and KA was 512 ± 330 days. Follow-up time after KA was a mean of 369 (± 350) days. Primary technical success for KA was achieved in all patients.

### Adverse Events

Four (8.3%) AEs were reported following KA procedures. These included two (4.2%) cases of delayed wound healing, potentially associated with GAE. One patient developed a wound dehiscence four weeks post KA requiring a subsequent incision and drainage procedure. The second patient developed a small skin eschar seven weeks following KA which was closed with a staple. Both AEs resolved and were classified as a grade 3 complications [3]. Additional non-GAE related AEs reported in two (4.2%) other patients. One was a stitch abscess and the second was non-infected fluid discharge at the surgical incision, both were classified as grade 2 complications [3]. The remaining 44 (91.7%) KAs did not develop post procedure AEs. No hemorrhagic or vascular complications occurred.

There were no significant differences between mean age (64.3 ± 4.2 vs. 68.7 ± 10.7 years, P = 0.239), BMI (25.4 ± 2.67 vs. 27.9 ± 5.2, P = 0.324), number of arteries treated (2.0 ± 0.8 vs. 1.8 ± 0.7, P = 0.693), and time between GAE and KA (491.8 ± 416.5 vs 513.4 ± 326.6 days, P = 0.787) between groups with and without AEs following KA, respectively. There was no significant difference in type of artery (Descending genicular artery: P = 0.182, Superior medial artery: P = 0.852, Inferior medial artery: P = 0.543, Superior lateral artery: P = 0.649, Inferior lateral artery: P = 0.349) treated between groups with and without AEs following KA. The group with AE’s consisted of 3 patients (3/4 = 75.0%) with a KL score of 3 and 1 patient (1/4 = 25.0%) with a KL score of 4. Comparatively the group without AE’s reported 1 patient (1/442.3%) with a KL score of 1, 6 patients (6/44 = 13.6%) with a KL score of 2, 29 patients (29/44 = 65.9%) with a KL score of 3, and 8 patients (8/44 = 18.2%) with a KL score of 4. There was no significant difference between Kellgren–Lawrence score distribution between groups with and without AE’s following KA (P = 0.856). There was also no significant difference between type of embolic used, embosphere (n = 9, 18.8%) vs embozene (n = 39, 81.2%) (P = 0.999).

## Discussion

With the increasing adoption of GAE as a viable treatment option for knee pain associated with OA, there is a need to validate the safety of KA following GAE. KA remains the gold standard therapy for patients with symptomatic advanced stage knee OA. As minimally invasive therapies develop and show promise as viable options prior to KA, it is essential that these therapies do not compromise the outcomes or safety of subsequent KA. While there is no current literature supporting findings of skin or bone necrosis directly related to GAE, it can be hypothesized that by impeding blood flow a GAE could theoretically cause ischemia of the synovium, bone, or skin which would make healing after a KA more challenging.

This study’s findings show an overall low AE rate after KA, which are comparable with other published studies [12, 13]. In fact, these percentages are lower than the NIH published rate of AE reported in stand-alone KAs, which was 21% for any AE and 6% for serious AEs [13]. Wound healing AEs following KA were reported as 7.6% [12]. The AE rate in this study for KA following GAE is 8%, with only 4% (2 out of the 48) being classified as wound healing related. This suggests that GAE prior to KA does not increase the risk for AEs during and after KA.

The low incidence of tissue ischemia after KA or after GAE can be partly attributed to procedural technique. In both study centers a high volume of GAEs were performed with similar technique. Two key components are critical to avoid tissue ischemia, which could preclude KA in the future. First, the size of embolic particles must be optimally chosen in order to treat hypervascularity without embolizing too distal to cause target ischemia (e.g. skin loss, bone infarcts, etc.). As a result, permanent embolic agents ranging in size from 100 to 300 micron were used. Permanent embolic sizes less than 100 micron may theoretically cause higher AEs after KA, as a prior study showed nerve damage in two cases with 75 micron particles [14]. This assertion is speculative and was not directly supported by our findings, further research is needed to assess the link between particle size and post-GAE wound complications. Second, it is imperative to not over-embolize. Post-embolization angiograms demonstrate persistent flow in the first and second order branches of the genicular arteries, with resolution of hyperemia [15]. This embolic endpoint is different than other embolic procedures, such as uterine and prostate artery embolization [7].

A few limitations to this study exist. First, the sample size is relatively small, given the high number of KA’s performed worldwide. Even with a sample size of only 48 knees this is the largest study looking at the safety in patients undergoing KA after GAE. However, the small size of the AE group (n = 4) limits our statistical power. The study size is limited by the novelty of the GAE procedure, by patients electing for KA after GAE, and availability of patient records. However, while relatively small, this study is multi-institutional including 16 orthopedic surgeons and six interventional radiologists, which allows broader applicability of the findings. Second, since this was a retrospective study via review of the electronic medical records, very minor AEs may have not been recorded by the treating orthopedic surgeon. Third, efficacy of KA was not assessed in this study, as standardized scoring systems (e.g. WOMAC, KOOS) were not performed in patients after KA by their orthopedic surgeons. The scoring systems are widely used in research protocols, but infrequently used in general clinical practice.

Another limitation stems from the variability in the operating surgeon during both the GAE and the KA. In order to obtain a larger sample size we recruited data from 6 IR doctors performing GAE and 16 orthopedic surgeons performing KA’s at 10 different institutions. While this increased our sample size it also introduced variability which could have impacted our findings. We also want to address that this study spanned 5 years, 2019 to 2024, in which operation physicians could have experienced a learning curve impacting the efficacy and success of both GAE and KA post GAE which also could have impacted our results. An additional limitation arises from the inclusion of both TKA and partial KAs as that again introduces variability that could have affected our findings.

In conclusion, our findings show that AEs potentially associated to GAE for patients undergoing KA are low and minor. However, it is important to keep in mind that a direct relationship between GAE and AE’s during and after KA remain speculative in the absence of a control group and further studies. This study concludes that GAE should not be considered a contraindication for future KA and may serve as a bridge in those trying to defer KA to a future date.
